# Comparison of Different Mass Spectrometry Workflows for the Proteomic Analysis of Tear Fluid

**DOI:** 10.3390/ijms23042307

**Published:** 2022-02-19

**Authors:** Garrett Jones, Tae Jin Lee, Joshua Glass, Grace Rountree, Lane Ulrich, Amy Estes, Mary Sezer, Wenbo Zhi, Shruti Sharma, Ashok Sharma

**Affiliations:** 1 Center for Biotechnology and Genomic Medicine, Medical College of Georgia, Augusta University, Augusta, GA 30912, USA; garjones@augusta.edu (G.J.); talee@augusta.edu (T.J.L.); josglass@augusta.edu (J.G.); grountree@augusta.edu (G.R.); wzhi@augusta.edu (W.Z.); shsharma@augusta.edu (S.S.); 2 Department of Ophthalmology, Medical College of Georgia, Augusta University, Augusta, GA 30912, USA; lulrich@augusta.edu (L.U.); aestes@augusta.edu (A.E.); msezer@augusta.edu (M.S.); 3 Department of Population Health Sciences, Medical College of Georgia, Augusta University, Augusta, GA 30912, USA

**Keywords:** tear film, proteomics, biomarkers, dry eye disease, ocular surface, mass spectrometry

## Abstract

The tear film is a multi-layer fluid that covers the corneal and conjunctival epithelia of the eye and provides lubrication, nutrients, and protection from the outside environment. Tear fluid contains a high concentration of proteins and has thus been recognized as a potential source of biomarkers for ocular disorders due to its proximity to disease sites on the ocular surface and the non-invasive nature of its collection. This is particularly true in the case of dry eye disease, which directly impacts the tear film and its components. Proteomic analysis of tear fluid is challenging mainly due to the wide dynamic range of proteins and the small sample volumes. However, recent advancements in mass spectrometry have revolutionized the field of proteomics enabling unprecedented depth, speed, and accuracy, even with small sample volumes. In this study using the Orbitrap Fusion Tribrid mass spectrometer, we compared four different mass spectrometry workflows for the proteomic analysis of tear fluid collected via Schirmer strips. We were able to establish a method of in-strip protein digestion that identified >3000 proteins in human tear samples from 11 healthy subjects. Our method offers a significant improvement in the number of proteins identified compared to previously reported methods without pooling samples.

## 1. Introduction

The tear film is a 2–6 µm stratified fluid, composed of mucoaqueous and lipid layers, that covers the corneal and conjunctival epithelia of the ocular surface. The mucoaqueous layer contains mucin and aqueous components, with mucin concentration decreasing along a gradient from the epithelium towards the aqueous layer. The mucins of the innermost layer extend large hydrophilic glycans to form the ocular surface glycocalyx [1]. The outermost layer of the tear film is a thin lipid layer that prevents evaporation of the mucoaqueous layer [2,3]. Proteins and lipids of the tear film safeguard the eye through anti-inflammatory, antioxidant, and antimicrobial activities [4,5,6,7]. Under healthy conditions, tears also provide nourishment to the apical epithelial cells and lubrication to the ocular surface to remove debris and prevent abrasion [2]. Insufficient secretion of the aqueous or lipid components of tears, laser eye surgery, and inflammatory pathologies, such as Sjögren’s syndrome, can result in tear film disruption, leaving the eye more vulnerable to infection and inflammation [8,9]. Thus, the integrity of this film is necessary for maintaining normal vision and preventing ocular damage.

Dry eye disease (DED) is a multifactorial disease of the ocular surface in which hyperosmolarity and instability of the tear film trigger inflammation, inducing a positive feedback loop of disease progression that eventually leads to corneal abrasions and ulceration [10]. Although the disease impacts both vision and quality of life, diagnosis of DED remains highly subjective and difficult due to an inconsistent correlation between symptom onset and clinical signs, as well as the absence of definitive diagnostic biomarkers [11,12,13]. Tear proteins are a potential source for identification of biomarkers for diagnosing and monitoring DED and its underlying pathophysiology [14,15,16,17,18]. 

**Figure 1:**
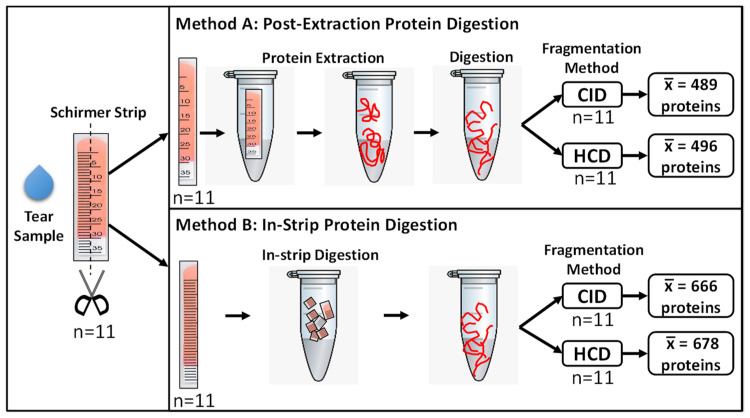
Comparison of two extraction methods (Method A and B) and two fragmentation methods (CID and HCD) for tear fluid processing. Tear fluid was collected using Schirmer strips (*n* = 11), and each strip was cut longitudinally into two equal parts. In Method A, proteins were first extracted, and Schirmer strips were removed by filter-aided centrifugation prior to digestion. In Method B, Schirmer strips were cut into 5 mm pieces, and in-strip protein digestion was performed. Digested products from each method then underwent LC–MS/MS analysis using both collision-induced dissociation (CID) and higher-energy collisional dissociation (HCD) fragmentation techniques. The average number of unique proteins ($\bar{x}$) identified per sample using each workflow is displayed.

In the past, proteomic analysis of tear fluid was challenging due to its small volume. However, recent technological advancements in mass spectrometry, such as the Orbitrap ion trap mass analyzer, have increased the depth, speed, and sensitivity of protein identification [17,19,20]. Beam-type collisional dissociation fragmentation techniques, such as collision-induced dissociation (CID) and higher-energy collisional dissociation (HCD), allow for high confidence identification of peptides, as well as their potential post-translational modifications [21]. These improvements allow for the precise and high-throughput proteomic profiling of small sample volumes, making it the ideal method for the identification of proteins and peptides in the tear film. To provide high-value proteomic data from tears, rigorous testing of tear protein processing methods coupled with mass spectrometry analysis is required. In this study, we compared four different workflows for the analysis of the tear proteome using mass spectrometry (Figure 1).

## 2. Results

Tear samples were collected from 11 healthy subjects using Schirmer strips. The demographic information of the subjects is detailed in Table 1. A total of four analysis workflows, including two distinct protein digestion methods (post-extraction protein digestion and in-strip protein digestion) and two fragmentation techniques (CID and HCD), were compared.

### 2.1. In-Strip Protein Digestion Identifies More Proteins Than Post-Extraction Protein Digestion

**Figure 2:**
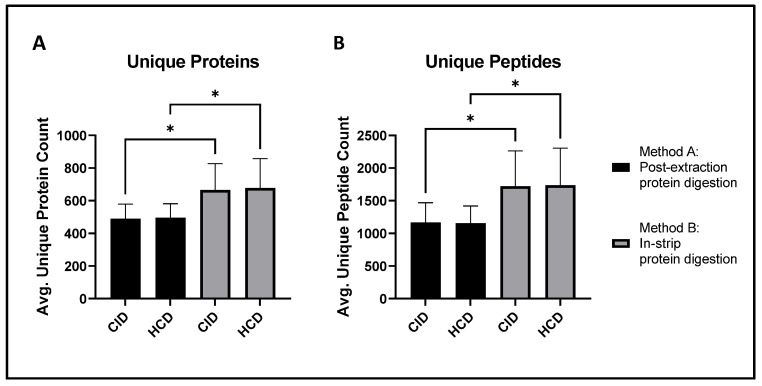
In-strip protein digestion (Method B) provides a significant increase in both protein and peptide yield compared to post-extraction protein digestion (Method A) in tear fluid samples. The average protein and peptide counts per sample identified in all four workflows were compared via two-way ANOVA with Tukey’s correction. (**A**) Method B identified more proteins than Method A when paired with both CID and HCD fragmentation. (**B**) In-strip protein digestion also identified more peptides than post-extraction digestion following both CID and HCD fragmentation. Results are expressed as means ± SD; *n* = 11/group; * *p*-value <0.05.

Post-extraction protein digestion (Method A) identified an average of 489 ± 90 unique proteins per sample after CID fragmentation and 496 ± 85 unique proteins per sample after HCD fragmentation. In-strip protein digestion (Method B) identified an average of 666 ± 161 unique proteins per sample when CID fragmentation was used and 678 ± 180 per sample when HCD fragmentation was used (Figure 2A). Thus, in-strip protein digestion allows for the identification of significantly more proteins using either CID (*p*-value = 0.0160) or HCD (*p*-value = 0.0203) fragmentation. Similar trends were observed at the peptide level (Figure 2B). In-strip protein digestion identified significantly more unique peptides when paired with both CID (1720 ± 543 vs. 1167 ± 302; *p*-value = 0.0266) and HCD (1735 ± 567 vs. 1155 ± 264; *p*-value = 0.0185) fragmentation. There were no significant differences between the number of unique proteins or peptides identified via HCD and CID fragmentation when the same protein digestion method was used. 

### 2.2. Proteomic Profiling of Tear Samples

**Figure 3:**
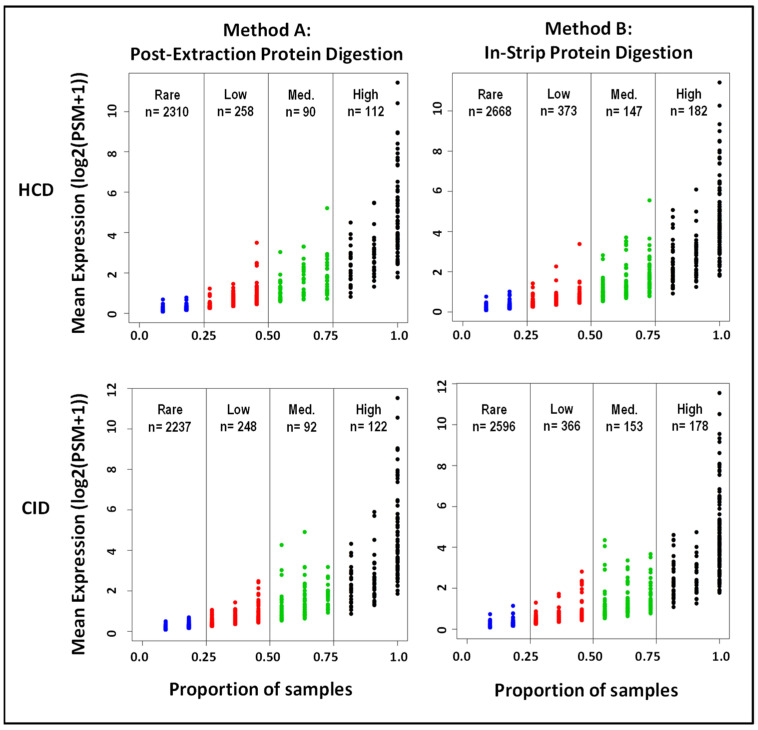
Distribution of mean protein expression by sample proportion in the four different workflows. Peptide–spectrum matches (PSMs) from the 11 tear samples were log2 transformed for each digestion and fragmentation method performed to compare differences in mean protein expression between workflows. Further, proteins detected in the 11 samples were proportionally assessed and subdivided into four categories based on trends of detection for each method: High (shown in black; detected in >75% of samples), Medium (shown in green; detected in 50–75%), Low (shown in red; detected in 25–50%), and Rare (shown in blue; detected in 5–25%).

The average protein levels, quantified by the number of peptide–spectrum matches (PSMs), were plotted against the proportion of samples in which they were detected, showing a positive correlation (Figure 3). The total unique proteins identified using the four different workflows are compared in Table 2. Based on their relative occurrence, these proteins were categorized into four groups: high (present in >75% of samples), medium (present in 50–75% of samples), low (present in 25–50% of samples), or rare (present in 5–25% of samples). A total of 3370 unique proteins were identified in the tear samples subjected to in-strip protein digestion and HCD fragmentation (high, *n* = 182; medium, *n* = 147; low, *n* = 373; and rare, *n* = 2668). Since this workflow identified the greatest number of unique proteins, these 3370 unique proteins were further examined using bioinformatics approaches to determine the characteristics of the proteome of human tear film. The 50 most abundant proteins detected using in-strip protein digestion and HCD fragmentation are listed in Table 3.

### 2.3. Major Protein Families Identified in Human Tear Samples

Several major protein families are over-represented in the proteomic profile of human tear samples, including immunoglobulins (61 proteins), keratins (26 proteins), complements (17 proteins), myosins (15 proteins), apolipoproteins (11 proteins), heat shock proteins (10 proteins), protein s100 family (9 proteins), annexins (8 proteins), 14-3-3 proteins (7 proteins), cystatins (6 proteins), and peroxiredoxins (5 proteins). Of these families, immunoglobulins had the greatest number of highly-expressed proteins (17 proteins). All identified proteins within these families are listed in Table 4.

### 2.4. Gene Ontology Analyses of Differentially Expressed Proteins in Tear Fluid

Using in-strip protein digestion and HCD fragmentation, a total of 329 proteins were detected in at least 50% of the tear samples. Gene ontology (GO) analysis was performed to determine biological processes, cellular compartments, and molecular functions associated with these proteins. The most highly enriched GO terms are displayed in Table 5.

### 2.5. Interaction Network Analyses

**Figure 4:**
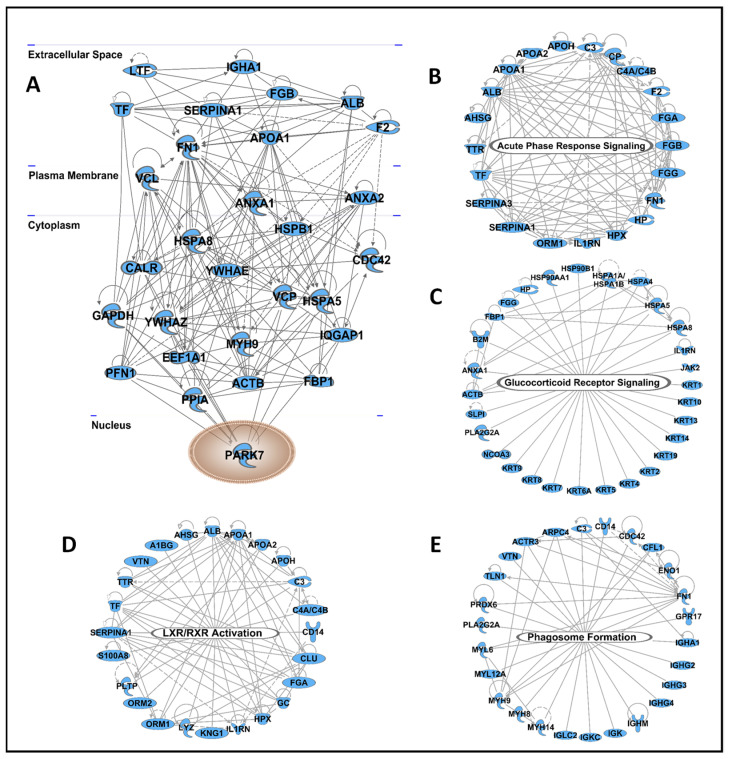
Network analyses revealed tear proteins with the highest level of interactions and four canonical pathways enriched in tear proteins. All 329 proteins detected in at least 50% of the samples were analyzed using Ingenuity Pathway Analysis software. (**A**) Proteins that showed the highest level of protein–protein interactions are depicted by cellular location. A total of four canonical pathways were highly enriched in tear proteins, including acute phase response signaling (**B**), glucocorticoid receptor signaling (**C**), LXR/RXR signaling (**D**), and phagosome formation (**E**).

The 329 proteins detected in at least 50% of the tear samples were further analyzed using Ingenuity Pathway Analysis (IPA) to generate interaction networks and visualize major hubs. A total of 29 proteins connected to more than 15 nodes were considered major hubs of the interaction network (Figure 4A). The proteins with the highest level of interaction with other proteins include two 14-3-3 proteins (*YWHAZ* and *YWHAE*), fibronectin (*FN1*), three heat shock proteins (*HSPA8*, *HSPA5*, and *HSPB1*), two annexins (*ANXA1* and *ANXA2*), and alpha-1-antitrypsin (*SERPINA1*). IPA analyses also revealed four major canonical pathways highly enriched in the tear proteins, including acute phase response signaling, glucocorticoid receptor signaling, LXR/RXR activation, and phagosome formation (Figure 4B–E).

## 3. Discussion

Advancements in mass spectrometry over the last two decades, including the advent of Orbitrap and higher-energy collision dissociation, have increased yields of proteins and peptides from very small sample volumes, such as tear fluid. In this study, we compared four different workflows, consisting of two digestion and two fragmentation methods, to establish an optimized workflow for the proteomic analysis of human tear samples. While our in-strip protein digestion method produced a clear improvement in the number of identified proteins and peptides compared to pre-extraction digestion, both CID and HCD fragmentation produced nearly identical yields; however, HCD fragmentation provides additional information for identifying and assessing post-translational modifications, such as glycosylation [22]. Given the abundance of glycoproteins in tears [23], as well as the significant roles they play in maintaining ocular surface homeostasis [24], HCD fragmentation combined with in-strip digestion was selected as the optimal workflow for the proteomic analysis of tears; this will facilitate future glycoproteomic studies.

Using the selected workflow, a total of 3370 unique proteins were identified, with an average of 678 unique proteins per sample. This is an improved and more sensitive detection method compared to previously reported LC–MS/MS methods [17,25,26,27,28,29,30,31,32,33,34,35,36,37,38,39,40,41,42,43,44,45,46]. Aass et al. detected an average of 309 proteins per subject (pooling 2 strips collected from both eyes) using the same post-extraction protein digestion method, though only paired with CID fragmentation [29]. It is important to note that our workflow did not involve pooling multiple strips together to increase protein detection. Since ocular surface disorders are not always bilateral and each eye has distinct properties, this allows for the more accurate correlation between disease states and tear proteomic profiles [47]. Thus, our proposed workflow, including our in-strip protein digestion method, can be used in future studies attempting to identify tear proteomic biomarkers in ocular diseases. This is the first step in developing diagnostic and prognostic assays for clinical use.

GO and IPA analyses of data from our chosen workflow identified the most abundant proteins as members of the immunoglobulin, keratin, and complement families. Detecting these protein families in the tear fluid offers potential to identify biomarkers for DED and other ocular surface diseases due to the roles of such proteins in mediating immune function and barrier protection. In particular, immunoglobulins, which play a role in protecting the eye from pathogens, were the most abundant protein family identified in our analyses, with 61 proteins detected in the tear fluid. Immunoglobulin A (IgA) is known to be present in mucous membranes, and its high expression in tears has been consistently reported [48]. IgA is produced by acinar cells of the lacrimal gland before being secreted into the aqueous layer of the tear film, where the antibody acts to neutralize pathogens and prohibit their adhesion and invasion of cells at the ocular surface [49]. A recent study by McKay et al. found that differences in the distribution of immunoglobulin chains within tear fluid is associated with keratoconus, another ocular surface disease [48]. Thus, the diversity of Ig chains that were identified in most samples within this small cohort offers an opportunity for insight into different immune-mediated pathologies of the eye.

Seventeen complement family proteins were also present in the majority of tear samples from our chosen workflow. This finding supports recent evidence that low-level constitutive activation of the complement system is found in the cornea and tears of healthy individuals [50]. While the exact role of the complement system on the ocular surface is unknown, it has been suggested that immune or inflammatory responses may trigger an aggressive complement cascade defense [50]. Given the potential for inflammation-mediated damage, the interplay of the complement system on the ocular surface needs to be investigated further.

In addition to immunoregulatory families, the apolipoprotein family was highly represented in tear fluid, with 11 apolipoproteins identified in our analyses. Apolipoproteins A1 (*APOA1*) and A2 (*APOA2*) are produced primarily in the liver and are best known for carrying cholesterol as the constitutive proteins of high-density lipoprotein (HDL) [51,52]. In the meibomian gland, *APOA1* functions to reverse cholesterol transport, preventing the build-up of free cholesterol that can otherwise lead to meibomian gland dysfunction (MGD) [53]. Also, a previous study has shown that *APOA2* is upregulated in the tears of patients with diabetes [54]. 

Structural proteins of the ocular surface, such as keratins and myosins, are commonly found in tear fluid in both healthy and diseased conditions [55]. Keratins are produced by the meibomian gland and form a protective covering for the eye [56]. Myosins are ubiquitously expressed across cell types, and their cellular functions are diverse, including roles in polarization, movement, and exocytosis [57,58,59]. Specifically, myosin 6, found in highest abundance, is necessary for iris development [60]. In our analysis of human tear fluid with the chosen workflow, we were able to detect 26 keratin-family proteins and 15 myosin-family proteins.

Consistent with other studies, lactotransferrin (*LTF*), lipocalin-1 (*LCN1*), albumin (*ALB*), and prolactin-inducible protein (*PIP*) were the top four proteins detected with the highest levels in the tear film [61]. Lactotransferrin is a glycoprotein with iron-chelating properties that aid in the defense of the mucosa [62]. However, lactotransferrin has also demonstrated the ability to reduce dry-eye associated inflammation and prevent Herpes Simplex Virus (HSV) infection on the ocular surface [63]. Prolactin-inducible protein plays a role in increasing the placement of aquaporins in the apical cell membrane to provide lubrication to the ocular surface [64]. Lipocalin-1 is found primarily in the outermost lipid layer of the tear film, where it protects against desiccation. Thus, its levels in tear fluid are reduced in patients with deficient lacrimal secretion, such as Sjögren’s syndrome and other subtypes of aqueous deficient dry eye [65]. 

Tear proteins detected with the highest number of protein–protein interactions from IPA include 14-3-3 protein zeta/delta (*YWHAZ*), fibronectin (*FN1*), and heat shock protein family A members 8 (*HSPA8*) and 5 (*HSPA5*). High expression of the 14-3-3 family has been associated with Sjögren’s syndrome, although their exact role in the eye is still unclear [66]. Additionally, the expression of both *YWHAZ* and *HSPA8* has been shown to negatively correlate with fluorescein tear break-up time (FTBUT), suggesting these proteins may play a role in dry eye symptoms [66]. *HSPA5* levels have been shown to increase in tears in response to successful glaucoma treatment [66]. 

Since the tear film is an acellular biofluid, the majority of its protein and lipid contents are secreted by glandular and epithelial cells via exosomes. In our study, extracellular exosome and extracellular vesicle were highly enriched cellular components associated with tear proteins. Four major canonical pathways associated with the identified tear proteins were acute phase response signaling, glucocorticoid receptor signaling, liver X receptor/retinoid X receptor (LXR/RXR) signaling, and phagosome formation. The concentration of acute phase proteins (APPs) has been reported to change in response to inflammation [67]. Furthermore, the APP signaling pathway in the tear film is responsible for initiating immune cascades in the presence of inflammation, thus defining its role in the health of the ocular surface. A previous study found that changes in the tear fluid LXR/RXR signaling pathway are associated with uveitis [68].

Through our comparison of four different workflows, we have successfully identified a sensitive approach for discerning the proteomic profile of human tear fluid. Our findings further our understanding of the tear film by identifying pathways and protein families associated with the healthy tear film and its barrier integrity. Our in-strip protein digestion method coupled with HCD fragmentation may be adopted in future studies of human tear fluid to improve the diagnosis of ocular diseases and discern their underlying mechanisms.

## 4. Materials and Methods

### 4.1. Sample Collection

Tear samples were collected from 11 healthy subjects at the Department of Ophthalmology, Medical College of Georgia at Augusta University. The demographic information of the subjects is shown in Table 1. The study was approved by the Institutional Review Board at Augusta University (IRB Project ID# 1458143-12, Approval Date: 13 July 2021). Schirmer strips (TearFlo, HUB Pharmaceuticals, Scottsdale, AZ, USA) were used to collect the tear samples because of their previously reported ability to collect a greater number of unique proteins than capillary tubes [69]. Also, patients generally prefer their use over capillary tubes, as Schirmer strips are safer and perceived as less invasive [18]. First, each Schirmer strip was folded to a 90° angle at the 0 mm mark. The rounded end of the strip was inserted into the lateral portion of the lower conjunctival sac of the right eye for 5 min. The subjects’ eyes remained closed while the strips were in place. Upon removal, the strips were cut in half lengthwise into two equal pieces, and each half was placed in a separate cryogenic vial (#5000-0020, Thermo Fisher Scientific, Waltham, MA, USA); each half was used separately for the two protein digestion methods, as described in 4.2. The samples were kept at −80 °C until processed further.

### 4.2. Protein Digestion

Two distinct protein digestion methods were compared. For each strip collected from a single subject, proteins from one half of the Schirmer strip were extracted prior to protein digestion, as previously described (Method A) [29], while the other half was subjected to our newly developed method of in-strip protein digestion (Method B) (Figure 1). Thus, there were 11 biological replicates in each digestion group. For post-extraction protein digestion, 450 µL of 100 mM ammonium bicarbonate buffer with 50 mM sodium chloride (Sigma-Aldrich, St. Louis, MO, USA) was added to the strip and mixed for 4 h at 25 °C. The samples were then centrifuged in a centrifugal filter unit (#74-3840, Harvard Apparatus, Holliston, MA, USA) at 7500 rpm for 5 min, after which the strip was removed. The extracted proteins were lyophilized and re-dissolved in 60 µL of 8 M urea in 50 mM Tris–HCl (pH 8) (Sigma-Aldrich, St. Louis, MO, USA). For in-strip protein digestion, the Schirmer strip halves were first lyophilized to dryness. Each strip was then cut into 5 mm × 2.5 mm pieces, and 120 µL of 8 M urea in 50 mM Tris–HCl (pH 8) was added. Both sets of samples were reduced with 10 mM dithiothreitol, alkylated using 55 mM iodoacetamide and digested using MS-grade trypsin (#90057, Thermo Scientific, Waltham, MA, USA) at a 1:20 trypsin to protein ratio (*w*/*w*) overnight at 37 °C. Digested peptides were cleaned using C18 spin columns (#744101, Harvard Apparatus, Holliston, MA, USA) and then lyophilized before being analyzed on the Orbitrap Fusion Tribrid mass spectrometer coupled with an Ultimate 3000 nano-UPLC system (Thermo Fisher Scientific, Waltham, MA, USA).

### 4.3. LC–MS/MS

Four microliters of reconstituted peptides (in 2% acetonitrile with 0.1% formic acid) were loaded and washed on a Pepmap100 C18 trap (5 µm, 0.3 × 5 mm, Thermo Fisher Scientific, Waltham, MA, USA) at 20 µL/min using 2% acetonitrile in water (with 0.1% formic acid) for 10 min and then separated on a Pepmap100 RSLC C18 column (2.0 µm, 75 µm × 150 mm, Thermo Fisher Scientific, Waltham, MA, USA) using a gradient of 2 to 40% acetonitrile with 0.1% formic acid over 120 min at a flow rate of 300 nL/min and a column temperature of 40 °C. Eluted peptides were introduced into the Orbitrap Fusion MS via a nano-electrospray ionization source with a temperature of 300 °C and spray voltage of 2000 V by data-dependent acquisition in positive mode using Orbitrap MS analyzer for precursor scan at 120,000 FWHM from 400 to 2000 m/z and ion-trap MS analyzer for MS/MS scans in top speed mode (3 s cycle time) with dynamic exclusion settings (repeat count 1, repeat duration 15 s, and exclusion duration 30 s). All samples from Method A (*n* = 11) and Method B (*n* = 11) were subjected to LC–MS/MS twice to compare the efficacy of two common fragmentation methods, CID and HCD, using a normalized collision energy of 30%. 

### 4.4. Protein Identification and Analysis

Raw MS data were processed via the Proteome Discoverer software (version 2.2, Thermo Fisher Scientific, Waltham, MA, USA) and submitted for SequestHT search against the SwissProt human database. SequestHT search parameters were set as 10 ppm precursor and 0.6 Da product ion tolerance with static carbidomethylation (+57.021 Da) for cysteine and dynamic oxidation for methionine (+15.995 Da). The percolator peptide-spectrum matching (PSM) validator algorithm was used for PSM validation. Proteins unable to be distinguished based on the database search results were grouped to satisfy the principles of parsimony. A protein report was generated containing the identities and number of PSM for each protein group, which were further utilized for spectral counting based semi-quantitative analysis.

### 4.5. Statistical Analysis

The average protein and peptide counts per sample identified in all four workflows were compared via two-way ANOVA, and subsequent pairwise comparisons were performed using Tukey’s multiple comparison test in order to control the family wise error rate [70]. The PSM values generated from LC–MS/MS analysis were log2 transformed to remove skewness. Proteins were allocated into groups using their mean expression level and detection in a proportion of samples. The ubiquitous proteins (detected in more than 50% of samples) were examined in further detail. The proteins were associated with biological processes, molecular functions, and cellular components using gene ontology (GO) enrichment analyses. All statistical analyses were performed using R version 4.0.3. Further, network analyses were performed using Ingenuity Pathway Analysis (IPA) to visualize the interactions between these proteins and identify the major hubs.

## 5. Conclusions

Multiple studies have shown the potential of tear proteomics for the discovery of diagnostic and prognostic biomarkers of several ocular and systemic diseases, including dry eye disease, pterygium, keratoconus, glaucoma, diabetic retinopathy, cancer, systemic sclerosis, and cystic fibrosis [20,71,72,73]. Due to its wide array of possible applications, an optimized workflow for tear processing holds immense translational potential. In this study, we have compared different mass spectrometry workflows and established a more sensitive and reliable method of tear protein detection and analysis that can be used for future tear proteomic biomarker research. 

## Figures and Tables

**Table 1:** Demographic characteristics of healthy subjects included in this study.

Sample ID	Age	Sex	Race
S1	27	F	White
S2	50	F	Other
S3	30	F	Other
S4	22	M	White
S5	40	M	Other
S6	27	F	White
S7	21	F	White
S8	45	M	Other
S9	25	F	White
S10	26	F	White
S11	22	F	White

**Table 2:** Total number of unique proteins identified in tear samples using four different workflows.

Number of Unique Proteins Identified	Method A		Method B	
	CID	HCD	CID	HCD
High (detected in >75% of samples)	122	112	178	182
Medium (detected in 50–75% of samples)	92	90	153	147
Low (detected in 25–50% of samples)	248	258	366	373
Rare (detected in 5–25% of samples)	2237	2310	2596	2668
Total	2699	2770	3293	3370

**Table 3:** Top 50 proteins identified in tear samples using in-strip protein digestion and HCD fragmentation.

S. No.	UniProt ID	Gene Symbol	Description	Mean PSM
1	P02788	*LTF*	Lactotransferrin	2802.91
2	P31025	*LCN1*	Lipocalin-1	1300.64
3	P02768	*ALB*	Albumin	874.18
4	P12273	*PIP*	Prolactin-inducible protein	572.46
5	P01876	*IGHA1*	Immunoglobulin heavy constant alpha 1	386.19
6	P61626	*LYZ*	Lysozyme C	380.45
7	P01833	*PIGR*	Polymeric immunoglobulin receptor	275.36
8	Q9GZZ8	*LACRT*	Extracellular glycoprotein lacritin	263.91
9	P01834	*IGKC*	Immunoglobulin kappa constant	230.09
10	P0DOX2	*IGA2*	Immunoglobulin alpha-2 heavy chain	212.87
11	P25311	*AZGP1*	Zinc-alpha-2-glycoprotein	203.00
12	P0DOX7	*IGK*	Immunoglobulin kappa light chain	189.36
13	P01036	*CST4*	Cystatin-S	185.90
14	O75556	*SCGB2A1*	Mammaglobin-B	137.72
15	Q16378	*PRR4*	Proline-rich protein 4	130.54
16	P01037	*CST1*	Cystatin-SN	115.40
17	P19013	*KRT4*	Keratin, type II cytoskeletal 4	100.11
18	Q99935	*OPRPN*	Opiorphin prepropeptide	97.72
19	P60709	*ACTB*	Actin, cytoplasmic 1	97.63
20	P06733	*ENO1*	Alpha-enolase	96.63
21	P04083	*ANXA1*	Annexin A1	95.45
22	Q9UGM3	*DMBT1*	Deleted in malignant brain tumors 1 protein	86.00
23	P01024	*C3*	Complement C3	80.54
24	P02787	*TF*	Serotransferrin	78.72
25	B9A064	*IGLL5*	Immunoglobulin lambda-like polypeptide 5	76.11
26	Q8N3C0	*ASCC3*	Activating signal cointegrator 1 complex subunit 3	73.45
27	P0DOX5	*IGG1*	Immunoglobulin gamma-1 heavy chain	73.00
28	P0DOY2	*IGLC2*	Immunoglobulin lambda constant 2	70.18
29	P13646	*KRT13*	Keratin, type I cytoskeletal 13	70.11
30	P08727	*KRT19*	Keratin, type I cytoskeletal 19	66.00
31	P13647	*KRT5*	Keratin, type II cytoskeletal 5	56.00
32	P09211	*GSTP1*	Glutathione S-transferase P	51.18
33	P68032	*ACTC1*	Actin, alpha cardiac muscle 1	48.10
34	P09228	*CST2*	Cystatin-SA	47.71
35	P01860	*IGHG3*	Immunoglobulin heavy constant gamma 3	47.14
36	P14618	*PKM*	Pyruvate kinase PKM	46.54
37	P01591	*JCHAIN*	Immunoglobulin J chain	46.00
38	P98160	*HSPG2*	Heparan sulfate proteoglycan core protein	45.72
39	P07355	*ANXA2*	Annexin A2	44.81
40	P0DOX6	*IGM*	Immunoglobulin mu heavy chain	44.28
41	P21980	*TGM2*	Protein-glutamine gamma-glutamyltransferase 2	43.00
42	P01871	*IGHM*	Immunoglobulin heavy constant mu	41.90
43	P30740	*SERPINB1*	Leukocyte elastase inhibitor	40.54
44	P98088	*MUC5AC*	Mucin-5AC	40.33
45	P02538	*KRT6A*	Keratin, type II cytoskeletal 6A	40.33
46	P00450	*CP*	Ceruloplasmin	40.18
47	P00352	*ALDH1A1*	Aldehyde dehydrogenase 1A1	40.18
48	P01861	*IGHG4*	Immunoglobulin heavy constant gamma 4	40.00
49	P01859	*IGHG2*	Immunoglobulin heavy constant gamma 2	37.90
50	P08729	*KRT7*	Keratin, type II cytoskeletal 7	37.14

**Table 4:** Major protein families identified in human tear samples.

Families	Group	Count	Proteins					
Immunoglobulin	High	**17**	*IGHV4-59*	*IGHV5-51*	*JCHAIN*	*IGHA1*	*IGHM*	*IGKC*
			*IGKV1D-33*	*IGHG2*	*IGLV3-9*	*IGKV1-8*	*IGLC2*	*IGK*
			*IGKV2D-29*	*IGKV3-15*	*IGHV3-7*	*IGKV4-1*	*IGLL5*	
	Medium	8	*IGA2*	*IGKV3-20*	*IGHV6-1*	*IGG1*	*IGHG3*	*IGHG4*
			*IGM*	*IGLV1-47*				
	Low	7	*IGKV3D-11*	*IGKV3-11*	*IGHV3-15*	*IGHV3-9*	*IGHA2*	*IGHG1*
			*IGKV3D-20*					
	Rare	29	*IGHV1-69D*	*IGKV1D-8*	*IGHV3-72*	*IGHV3-74*	*IGHV3-49*	*IGHV3-33*
			*IGKV6D-21*	*IGLC1*	*IGLV1-40*	*IGLV1-44*	*IGLV6-57*	*IGSF22*
			*IGHV3-64D*	*IGHV1-18*	*IGHV2-26*	*IGHV3-64*	*IGD*	*IGHV4-28*
			*IGHV5-10-1*	*IGKV5-2*	*IGKV3D-15*	*IGKV1-16*	*IGKV1-17*	*IGKV1-6*
			*IGKV1D-13*	*IGLV3-19*	*IGLL1*	*IGSF10*		
Keratin	High	7	*KRT10*	*KRT9*	*KRT1*	*KRT2*	*KRT13*	*KRT19*
			*KRT4*					
	Medium	5	*KRT14*	*KRT5*	*KRT7*	*KRT6A*	*KRT8*	
	Low	2	*KRT18*	*KRT3*				
	Rare	12	*KRT15*	*KRT82*	*KRT85*	*KRT78*	*KRT31*	*KRT34*
			*KRTAP5-1*	*KRT17*	*KRT23*	*KRT83*	*KRT86*	*KRT36*
Complement	High	2	*C3*	*CFB*				
	Medium	2	*C4A*	*CD55*				
	Low	3	*C1QTNF4*	*C1QB*	*CFH*			
	Rare	10	*CFHR1*	*C1QTNF2*	*C9*	*C1RL*	*C1S*	*C4B*
			*C7*	*CFHR5*	*CFI*	*CR1*		
Myosin	High	4	*MYL6*	*MYH14*	*MYL12A*	*MYH8*		
	Medium	1	*MYH9*					
	Low	3	*MYO3A*	*MYH13*	*MYH10*			
	Rare	7	*MYH15*	*MYL1*	*MYLK4*	*MYLK*	*MYH2*	*MYH7*
			*MYH7B*					
Apolipoprotein	High	2	*APOA1*	*APOA2*				
	Medium	0						
	Low	2	*APOD*	*APOB*				
	Rare	7	*APOA4*	*APOC3*	*APOL1*	*APOC2*	*APOE*	*APOF*
			*LPA*					
Heat shock	High	4	*HSPA1A*	*HSPB1*	*HSP90AA1*	*HSPA8*		
	Medium	1	*HSPA4*					
	Low	1	*HSP90AB1*					
	Rare	4	*HSP90AA2P*	*HSPA13*	*HSPA1L*	*TRAP1*		
Protein s100	High	4	*S100A11*	*S100A4*	*S100A8*	*S100A9*		
	Medium	0						
	Low	0						
	Rare	5	*S100A10*	*S100A14*	*S100A7L2*	*S100A2*	*S100A7*	
Mucin	High	1	*MUC5AC*					
	Medium	0						
	Low	1	*MUC16*					
	Rare	6	*MUC12*	*MUC17*	*MUC19*	*MUC2*	*MUC5B*	*MUC6*
Annexin	High	5	*ANXA1*	*ANXA2*	*ANXA5*	*ANXA3*	*ANXA4*	
	Medium	1	*ANXA11*					
	Low	0						
	Rare	2	*ANXA10*	*ANXA8L1*				
14-3-3	High	4	*YWHAB*	*YWHAZ*	*YWHAE*	*SFN*		
	Medium	1	*YWHAG*					
	Low	1	*YWHAQ*					
	Rare	1	*YWHAH*					
Cystatin	High	4	*CSTB*	*CST3*	*CST4*	*CST1*		
	Medium	1	*CST2*					
	Low	1	*CST5*					
	Rare	0						
Peroxiredoxin	High	4	*PRDX1*	*PRDX5*	*PRDX6*	*PRDX2*		
	Medium	0						
	Low	0						
	Rare	1	*PRDX4*					

**Table 5:** Gene Ontology (GO) enrichment analysis of selected tear proteins.

GO ID	GO Term	# of Proteins	*p*-Value
**Biological Processes**			
GO:0052548	Regulation of endopeptidase activity	42	1.43 × 10^−22^
GO:0006508	Proteolysis	79	3.50 × 10^−20^
GO:0006950	Response to stress	122	6.75 × 10^−19^
GO:0051336	Regulation of hydrolase activity	58	6.83 × 10^−19^
GO:0009605	Response to external stimulus	90	8.18 × 10^−14^
GO:0006952	Defense response	66	1.67 × 10^−13^
GO:0007010	Cytoskeleton organization	59	1.96 × 10^−13^
GO:0042592	Homeostatic process	65	4.36 × 10^−12^
GO:0010941	Regulation of cell death	61	8.42 × 10^−12^
GO:0098542	Defense response to other organism	44	1.09 × 10^−09^
GO:0009617	Response to bacterium	35	1.21 × 10^−09^
GO:0006915	Apoptotic process	62	1.26 × 10^−09^
GO:0006954	Inflammatory response	36	2.09 × 10^−09^
GO:0051050	Positive regulation of transport	38	4.16 × 10^−09^
GO:0022610	Biological adhesion	51	1.20 × 10^−08^
GO:0006793	Phosphorus metabolic process	76	1.41 × 10^−08^
**Cellular Components**			
GO:0070062	Extracellular exosome	224	1.41 × 10^−152^
GO:1903561	Extracellular vesicle	224	1.74 × 10^−148^
GO:0005576	Extracellular region	244	1.35 × 10^−104^
GO:0072562	Blood microparticle	36	4.77 × 10^−34^
GO:0101002	Ficolin-1-rich granule	34	1.36 × 10^−27^
GO:0070161	Anchoring junction	55	3.09 × 10^−21^
GO:0005764	Lysosome	42	1.89 × 10^−14^
GO:0005773	Vacuole	44	6.14 × 10^−14^
GO:0030054	Cell junction	70	1.84 × 10^−11^
**Molecular Functions**			
GO:0061135	Endopeptidase regulator activity	31	2.56 × 10^−23^
GO:0061134	Peptidase regulator activity	33	3.18 × 10^−23^
GO:0050839	Cell adhesion molecule binding	44	1.80 × 10^−20^
GO:0045296	Cadherin binding	35	4.47 × 10^−20^
GO:0030234	Enzyme regulator activity	63	1.35 × 10^−18^
GO:0008289	Lipid binding	36	1.77 × 10^−09^

## Data Availability

The mass spectrometry proteomics data have been deposited to the ProteomeXchange Consortium via the PRIDE partner repository with the dataset identifier PXD030990).
